# Heart Disease : Its Causes and Treatment

**Published:** 1875-07

**Authors:** E. H. Gibbs


### HEART DISEASE; ITS CAUSES AND TREATMENT.

BY E. H. GIBBS, M.D.

Post-mortem examinations of sixty persons supposed to have died of "heart disease," in Paris, disclosed the fact, that but three of the number had suffered from serious disorders of that organ. Fifty-seven of the sixty deaths were caused by congestion of the brain, or lungs; and only three from diseases of the heart; that is, only one in twenty were ever seriously affected by the diseases of which they were reported to have died. This is not claimed as the exact ratio of errors in reported deaths from heart diseases in the United States, but it probably approximates these figures. The reported cause of sudden death may usually be attributed more to the apathy than to the ignorance of the physician who may be called upon to determine it.

The relatives and friends have met with a sudden bereavement, and demand the cause of death. They can hardly wait to have the question settled by an autopsy, even if they would permit it to be made. They know that certain organic diseases of the heart speedily destroy life, and to have their own views confirmed by a medical man is eminently satisfactory and consoling. On a casual view this seems proper enough, and entirely harmless; but in fact it is an unjustifiable deception, and produces a great amount of mental suffering, by propagating the belief that fatal diseases of the heart are very prevalent; whereas, in comparison with many other diseases, they are not. There is no community so small but has its victims of imaginary diseases, who are quick to listen to reports confirmatory of their worst fears.

There can be no philanthropy greater, no charity more noble, than to relieve the mental sufferings of these unfortunate people. This can be done effectually by furnishing them with correct information relating to the laws of health and disease, (particularly in regard to those diseases that hypochondriacs always suppose themselves afflicted with,) giving them such facts and statistics as will often allay their fears and restore them to *reason* and to health.

There are, probably, ten diseases most of them functional and curable that the imagination of the patient will generally locate in the heart. It follows then that if any ten supposed sufferers from heart disease, were to be subjected to careful examination, but one of the number would find his fears realized. When, moreover, it is considered that but few of those actually affected, die of these diseases, the number of real sufferers is reduced to a very small percentage.

Affections of the heart are far more common in mountainous countries than in localities nearer the sea level. But the fact that certain elevated regions are comparatively exempt, seems to indicate that there may be other causes beside rarity of the atmosphere to account for its greater prevalence in mountainous districts.

Santiago de Chile has a population of 160,000. It is only 4,000 feet above the sea level, and the summit of Aconcagua, just to the East, is nearly 20,000 feet above the city. There is no spot on earth where so large a percentage of the inhabitants have organic heart diseases as at Santiago. Still, the mortality from these causes is not great. The foreign population of the city seems to be almost exempt from these affections. It is probable that the habits of the natives have more to do with inducing these troubles than the location.

The climate is changeable; the dwellings are never warmed; and the clothing of the people is seldom changed to conform to changes of temperature.

The exemption of foreigners is doubtless owing to the better care they take of themselves. Violations of the laws of health will, sooner or later, produce derangement in the complicated machinery of the human body. No one can foresee in what form it will come; but it seldom, if ever, comes suddenly in the form of organic disease of the heart; this follows in the wake of some other affection; perhaps a mild form of chronic rheumatism. It is doubtful if organic heart disease ever exists without being heralded by rheumatic symptoms, generally of a mild type, because, perhaps, the severer forms receive prompt treatment. It becomes then the part of prudence to watch carefully, and promptly treat all such affections. A slight but persistent aching in a joint may be the monitor of serious trouble.

The treatment of rheumatism is often tedious and unsatisfactory. What will promptly relieve one case will have no effect on another, to all appearances similar. There are causes for this difference, which medical science may never discover. One patient is cured in an hour by "electricity;" another tries this remedy in vain, and is finally relieved by "sulphur vapor baths."

Others again have been cured by various medical compounds.

About seven years ago, a gentleman who had suffered for more than a year with severe rheumatism in the right shoulder, called on us for advice. He had tried in vain almost every remedy he could hear of. With a view of relieving the pain, and with no expectation of doing more, we injected a strong solution of acetate of morphine beneath the skin and near the point of severest pain, by means of a hypodermic syringe. He was very sick for about three hours, and vomited freely; but he had no more pain in the shoulder, and to this day has not felt the slightest twinge, and enjoys perfect health. We have since repeatedly employed the same treatment for rheumatism, but with no such satisfactory results. We conclude therefore that there is no *one* remedy applicable to all cases.

The treatment is empirical. If one course does not answer the purpose try another. The treatment adapted to each particular case is almost certain to be found by a reasonable exercise of patience and perseverance. The remedies which we have found the most successful are electricity, sulphur vapor bath, and friction or rubbing and kneading the parts affected.

As the May number of this Journal contains an article on "Spinal Irritation," we will only add, at present, our advice to look well and promptly to any indications of spinal disease, in order to prevent serious consequences.

It is comforting to know, that after all only a small proportion of sufferers from rheumatism, or spinal affections ever have organic diseases of the heart; and of those who do, but few die from those diseases. In most cases, the mind suffers far more than the body, and is in more need of treatment.

Nervous persons often fear to know the truth, lest it might be unfavorable.

They brood over their troubles watching suspiciously the vital operations, the beating of the heart, the pulse and respiration, imagining that they detect all sorts of irregularities, until by persistence in this course they frequently bring on the very troubles they dread most. At the first note of warning it is a duty we owe to ourselves, our families and our friends, to seek the best medical advice attainable. Such advice can come only from regularly educated and experienced physicians.

